# Supplementary material for: Analytical Pyrolysis of Pinus radiata and Eucalyptus globulus: Effects of Microwave Pretreatment on Pyrolytic Vapours Composition
Source: Polymers (Basel). 2023 Sep 17;15(18):3790. doi: 10.3390/polym15183790 (PMC10537089; doi:10.3390/polym15183790)
Supplement: Supplementary file 1 [file polymers-15-03790-s001.zip › polymers-2582477-supplementary.pdf]

polymers

MDPI

Article

Analytical Pyrolysis of *Pinus radiata* and *Eucalyptus globulus*: Effects of Microwave Pretreatment on Pyrolytic Vapours Composition

Diego Venegas-Vásconez [1,2], Luis E. Arteaga-Pérez [1,3], María Graciela Aguayo [1,4], Romina Romero-Carrillo [5], Víctor H. Guerrero [6], Luis Tipanluisa-Sarchi [7] and Serguei Alejandro-Martín [1,2,*]

1. Departamento de Ingeniería de Maderas, Universidad del Bío-Bío, Concepción, 4081112, Chile
2. Laboratorio de Cromatografía Gaseosa y Pirólisis Analítica, Universidad del Bío-Bío, Concepción, 4081112, Chile.
3. Laboratorio de Procesos Térmicos y Catalíticos, Universidad del Bío-Bío, Concepción, 4081112, Chile.
4. Centro de Biomateriales y Nanotecnología, Universidad del Bío-Bío, Concepción, 4081112, Chile.
5. Departamento de Química Analítica e Inorgánica, Facultad de Ciencias Químicas, Universidad de Concepción, Concepción, 4070371, Chile
6. Departamento de Materiales, Escuela Politécnica Nacional, Quito, 170525, Ecuador
7. Facultad de Mecánica, Escuela Superior Politécnica de Chimborazo, Riobamba, 060155, Ecuador

* Correspondence: salejandro@ubiobio.cl

Citation: Venegas-Vásconez, D.; Arteaga-Pérez, L. E.; Aguayo, M.G.; Romero-Carrillo, R.; Guerrero, V. H.; Tipanluisa-Sarchi, L.; Alejandro-Martín, S. Analytical Pyrolysis of *Pinus radiata* and *Eucalyptus globulus*: Effects of Microwave Pretreatment on Pyrolytic Vapours Composition. *Polymers* **2023**, *15*, x. https://doi.org/10.3390/xxxxx

Academic Editors: Chi-Hui Tsou and Chih-Yuan Tsou

Received: 12 August 2023
Revised: 10 September 2023
Accepted: 14 September 2023
Published: date

Copyright: © 2023 by the authors. Submitted for possible open access publication under the terms and conditions of the Creative Commons Attribution (CC BY) license (https://creativecommons.org/licenses/by/4.0/).

Table S1. Microwave pretreatment parameters for PR and EG.

**Sample**	**Microwave**	**1 min**			**2 min**			**3 min**			**5 min**		
		T_{avg}	T_{max}	W_{loss}	T_{avg}	T_{max}	W_{loss}	T_{avg}	T_{max}	W_{loss}	T_{avg}	T_{max}	W_{loss}
		**°C**	**°C**	**%**	**°C**	**°C**	**%**	**°C**	**°C**	**%**	**°C**	**°C**	**%**
PR	259	54,68 ± 1.25	64,41 ± 1.36	1,44 ± 0.13	63,02 ± 1.69	66,12 ± 2.15	4,01 ± 0.18	65,45 ± 1.76	69,61 ± 3.02	6,24 ± 0.35	73,35 ± 2.38	78,11 ± 1.89	7,61 ± 0.14
	462	78,33 ± 2.16	86,02 ± 0.95	3,76 ± 0.15	80,02 ± 2.41	86,29 ± 3.15	5,38 ± 0.09	88,57 ± 1.23	96,79 ± 2.15	7,86 ± 0.26	94,63 ± 3.01	106,79 ± 0.87	8,26 ± 0.16
	595	77,73 ± 1.87	84,79 ± 1.23	4,23 ± 0.24	84,57 ± 0.67	92,01 ± 2.89	6,25 ± 0.14	95,32 ±01.57	104,21 ± 2.54	8,97 ± 0.19	102,85 ± 2.68	115,72 ± 1.24	10,15 ± 0.17
	700	80,62 ± 1.93	93,50 ± 1.78	5,48 ± 0.23	87,45 ± 1.26	103,61 ± 2.46	7,31 ± 0.12	103,47 ± 2.47	120,32 ± 2.01	10,04 ± 0.82	115,17 ± 3.04	147,69 ± 1.56	11,06 ± 0.08
EG	259	42,78 ± 1.24	46,12 ± 0.49	0,69 ± 0.11	50,40 ± 1.47	57,70 ± 1.32	1,51 ± 0.04	50,98 ± 2.10	57,01 ± 1.23	2,63 ± 0.12	54,72 ± 0.16	59,71 ± 2.06	3,12 ± 0.41
	462	57,78 ± 1.47	65,69 ± 3.26	1,11 ± 0.05	68,68 ± 1.98	75,91 ± 1.52	2,14 ± 0.26	78,93 ± 1.56	85,98 ± 1.27	3,23 ± 0.15	90,31 ± 2.87	106,61 ± 1.78	3,72 ± 0.16
	595	72,63 ± 1.52	80,02 ± 1.89	1,33 ± 0.10	78,43 ± 2.01	84,89 ± 1.87	3,01 ± 0.13	83,67 ± 1.89	97,89 ± 1.96	4,12 ± 0.16	97,53 ± 2.16	109,10 ± 3.14	4,71 ± 0.32
	700	72,37 ± 1.71	79,21 ± 2.11	1,79 ± 0.15	94,82 ± 2.45	106,62 ± 1.59	3,47 ± 0.17	99,82 ± 2.03	118,02 ± 1.90	4,58 ± 0.14	101,98 ± 1.07	130,71 ± 2.79	5,17 ± 0.40

Table S2. Kinetic parameters and the characteristic temperatures of the thermogravimetric tests.

Bio-mass	α °C/min	Pretreatment W-min	$T_{initial}$ °C	T_{onset} °C	$T_{shoulder}$ °C	T_{peak} °C	T_{offset} °C	$Y_{shoulder}$ %	Y_{peak} %	Y_{offset} %	%Res %	$dY/dT_{shoulder}$ 10^{-3} s^{-1}	dY/dT_{peak} 10^{-3} s^{-1}
PR	5	WOT	28.84	231.42	309.25	345.61	367.09	61.84	89.01	22.54	13.53	-0.70	-1.06
		259 - 5	39.12	239.54	319.51	356.01	380.71	61.01	84.75	21.03	13.55	-0.70	-1.01
		700 - 5	36.24	251.64	326.00	358.07	386.93	62.04	68.02	25.01	15.05	-0.71	-0.82
	10	WOT	33.72	229.64	310.05	344.26	365.17	66.84	93.14	22.82	12.40	-0.73	-1.12
		259 - 5	38.88	244.71	319.03	355.84	377.86	67.25	81.45	25.19	16.89	-0.73	-0.97
		700 - 5	36.57	254.76	330.58	366.41	401.93	65.01	71.46	20.85	15.36	-0.73	-0.85
	20	WOT	33.81	228.56	309.85	343.86	365.76	66.87	91.85	24.17	8.26	-0.74	-1.08
		259 - 5	36.68	237.54	319.56	354.71	384.01	67.18	82.84	24.65	11.82	-0.74	-0.99
		700 - 5	39.21	249.16	327.17	366.85	399.61	68.23	69.13	21.83	13.89	-0.74	-0.83
EG	5	WOT	32.02	229.41	279.59	336.80	357.23	12.98	76.92	37.58	20.61	-0.48	-1.25
		259 - 5	50.88	233.76	297.32	349.17	369.33	15.05	77.58	40.21	22.53	-0.48	-1.15
		700 - 5	44.88	240.83	337.27	361.52	391.34	12.14	74.82	41.40	20.34	-0.46	-0.91
	10	WOT	33.48	225.39	278.34	338.17	355.14	12.42	77.42	37.33	19.12	-0.48	-1.27
		259 - 5	39.06	234.28	293.25	349.26	368.29	13.73	75.46	37.34	21.34	-0.48	-1.13
		700 - 5	50.41	240.36	339.13	360.27	396.32	9.88	73.18	25.98	18.30	-0.49	-0.94
	20	WOT	34.97	225.31	280.31	338.21	353.32	13.55	77.63	37.15	21.30	-0.47	-1.29
		259 - 5	48.01	234.76	291.46	348.40	369.37	11.59	77.78	37.19	19.48	-0.49	-1.20
		700 - 5	41.90	240.91	309.24	362.38	393.47	16.11	74.97	41.31	22.03	-0.44	-1.19

Table S3. Pyrolysis compounds of PR and EG (WOT, 259 - 700 W) (WE) and (WOE).

Family	Chemical compounds	PR %Area WE			PR %Area WOE			EG %Area WE			EG %Area WOE		
		WOT	259	700	WOT	259	700	WOT	259	700	WOT	259	700
Phenol	Creosol	7.10	6.68	5.79	6.45	6.00	5.81	3.01	2.66	3.01	2.23	2.22	2.58
Acid	Acetic acid	6.72	4.97	4.77	6.43	6.29	5.59	10.85	9.95	9.49	10.75	9.30	8.48
Phenol	2-Methoxy-4-vinylphenol	6.49	6.03	6.06	5.24	5.02	4.97	2.44	2.18	1.81	1.85	2.11	2.31
Phenol	Phenol, 2-methoxy-	5.84	5.20	4.85	4.20	3.71	3.97	1.69	1.70	1.70	0.94	1.12	1.25
Phenol	Phenol, 2-methoxy-4-(1-propenyl)-	5.32	5.13	4.27	0.73	4.80	5.73	0.61	0.55	0.58	0.31	0.21	0.21
Acid	Acetic acid, (acetyloxy)-	4.52	3.74	3.53	4.22	4.18	4.01	5.12	4.88	4.64	5.03	4.51	3.68
Alcohol	Acetol	4.00	5.72	6.83	4.87	5.56	5.72	4.35	4.98	5.93	3.53	4.43	4.61
Acid	Propanoic acid, 2-oxo-, methyl ester	3.61	2.72	1.32	3.02	2.92	1.26	2.69	2.40	2.30	2.78	2.85	1.64
Alcohol	2(5H)-Furanone	3.37	2.97	3.08	2.49	3.10	3.00	1.30	1.30	0.88	0.66	0.85	0.88
Ketone	2-Cyclopenten-1-one, 2-hydroxy-	3.09	3.50	3.67	3.21	3.95	4.10	2.93	3.06	3.56	3.14	3.57	4.00
Ketone	1,2-Cyclopentanedione, 3-methyl-	2.65	2.50	2.42	2.53	2.62	2.82	2.37	2.33	2.30	1.91	2.11	2.44
Alcohol	1,3-propanediol	2.63	2.63	2.63	2.43	2.66	2.70	0.70	0.75	0.78	0.53	0.74	0.67
Aldehyde	Succindialdehyde	2.51	2.28	2.10	2.12	2.37	3.14	2.14	2.15	2.27	1.28	1.70	2.32
Acid	Oxalic acid	2.33	2.33	2.15	2.28	1.57	1.51	5.48	5.29	4.60	4.69	4.41	3.48
Phenol	Phenol, 4-ethyl-2-methoxy-	2.28	1.99	1.63	2.89	2.32	2.14	0.69	0.71	0.69	1.42	0.57	0.75
Acid	Acetic acid ethenyl ester	2.07	1.85	1.67	2.20	2.16	1.87	1.86	1.98	2.12	1.40	1.78	2.35
Aldehyde	Furfural	1.75	1.99	2.21	2.06	2.18	2.30	2.64	2.74	2.81	2.06	2.25	3.00
Aldehyde	Methyl glyoxal	ND	2.14	2.71	3.64	4.25	4.18	3.12	3.47	3.65	2.77	3.80	4.25
Aldehyde	Benzaldehyde, 2,4-dihydroxy-6-methyl-	0.70	0.71	1.37	1.15	1.11	1.59	3.40	3.21	2.93	3.07	3.20	3.10
Phenol	Phenol, 2,6-dimethoxy-	ND	ND	ND	ND	ND	ND	5.13	4.91	4.54	5.12	4.74	4.60
Aldehyde	Hydroxyacetaldehyde	ND	ND	ND	ND	ND	ND	2.08	2.36	2.88	1.32	1.83	1.85

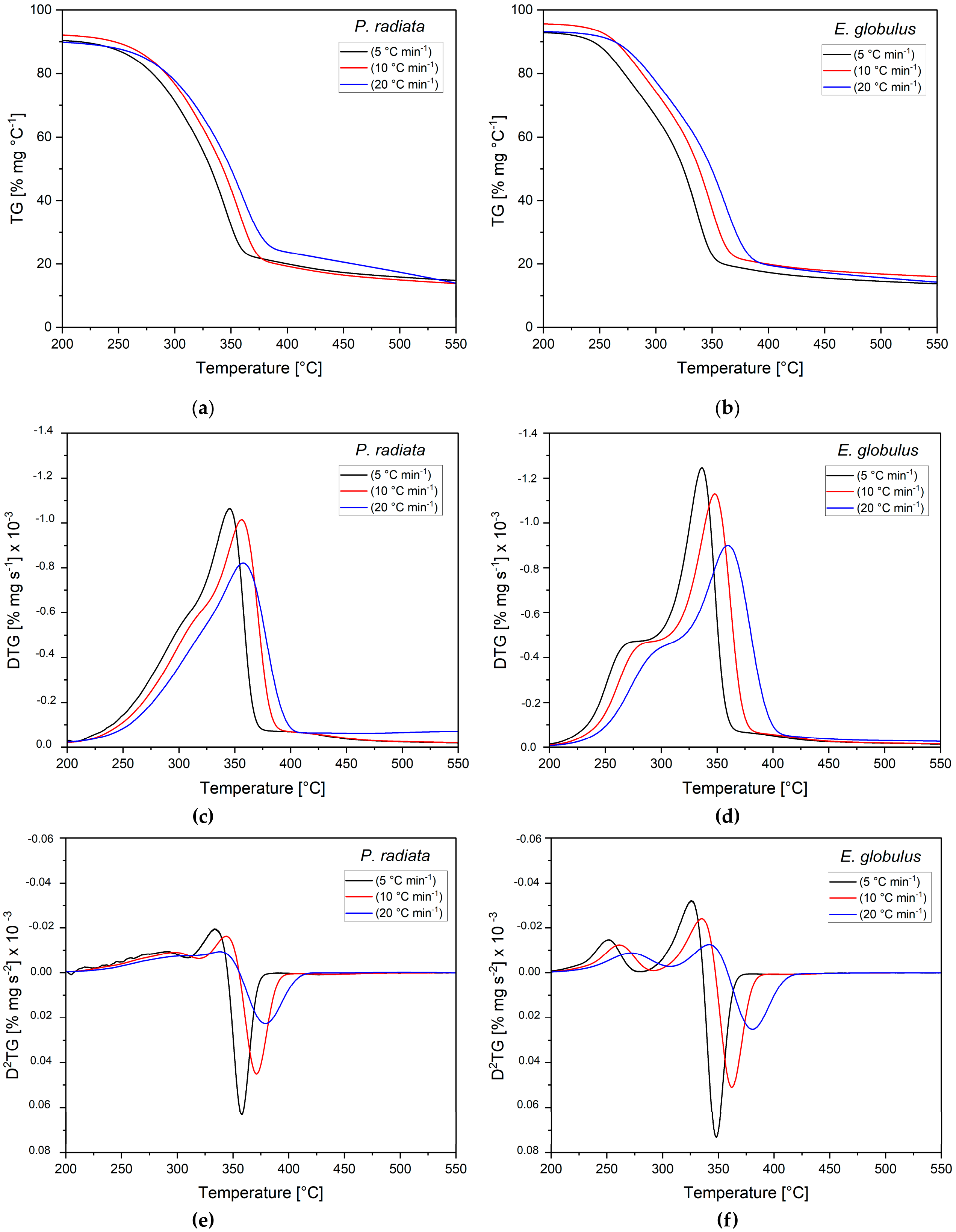

Figure S1. TG for a) *Pinus radiata* b) *Eucalyptus globulus*, DTG for c) *Pinus radiata* d) *Eucalyptus globulus* and D^2TG for e) *Pinus radiata* f) *Eucalyptus globulus* at 5, 10 and 20 °C min^{-1}.
